# Ursodeoxycholic acid exerts farnesoid X receptor-antagonistic effects on bile acid and lipid metabolism in morbid obesity

**DOI:** 10.1016/j.jhep.2014.12.034

**Published:** 2015-06

**Authors:** Michaela Mueller, Anders Thorell, Thierry Claudel, Pooja Jha, Harald Koefeler, Carolin Lackner, Bastian Hoesel, Guenter Fauler, Tatjana Stojakovic, Curt Einarsson, Hanns-Ulrich Marschall, Michael Trauner

**Affiliations:** 1 Hans Popper Laboratory of Molecular Hepatology, Division of Gastroenterology and Hepatology, Department of Internal Medicine III, Medical University of Vienna, Vienna, Austria; 2 Laboratory of Experimental and Molecular Hepatology, Division of Gastroenterology and Hepatology, Department of Medicine, Medical University of Graz, Graz, Austria; 3 Karolinska Institutet, Department of Clinical Science at Danderyds Hospital, Stockholm, Sweden; 4 Department of Surgery, Ersta Hospital, Stockholm, Sweden; 5 Core Facility for Mass Spectrometry, Medical University of Graz, Graz, Austria; 6 Institute of Pathology, Medical University of Graz, Graz, Austria; 7 Department of Vascular Biology and Thrombosis Research, Center for Physiology and Pharmacology, Medical University of Vienna, Vienna, Austria; 8 Clinical Institute of Medical and Chemical Laboratory Diagnostics, Medical University of Graz, Graz, Austria; 9 Karolinska Institutet, Department of Medicine, Karolinska University Hospital Huddinge, Stockholm, Sweden; 10 Institute of Medicine, Department of Molecular and Clinical Medicine, Sahlgrenska Academy, University of Gothenburg, Sweden

**Keywords:** BAs, bile acids, NAFLD, non-alcoholic fatty liver disease, UDCA, ursodeoxycholic acid, vWAT, visceral white adipose tissue, FXR, farnesoid X receptor, SCD, stearoyl-Coa desaturase, NASH, non-alcoholic steatohepatitis, TGs, triglycerides, FAs, fatty acides, CYP7A1, cholesterol 7α-hydroxylase, SHP, small heterodimer partner, FGF19, fibroblast growth factor 19, SREBP1c, sterol regulatory element-binding protein-1c, FASN, fatty acid synthase, VLDL, very low density lipoproteins, CDCA, chenodeoxycholic acid, CA, cholic acid, C4, 7α-hydroxy-4-cholesten-3-one, HMGCR, 3-hydroxy-3-methylglutaryl-CoA reductase, ABC, ATP-binding cassette, LDLR, low density lipoprotein receptor, MA, myristic acid, PA, palmitic acid, SA, stearic acid, OA, oleic acid, MTTP, microsomal triglyceride transfer protein, ApoB, apolipoprotein B, FATP1, fatty acid transport protein 1, nCEH, neutral cholesterol ester hydrolase, Non-alcoholic fatty liver disease, FGF19, 3-hydroxy-3-methylglutaryl-CoA reductase, Lipogenesis, Stearoyl-CoA desaturase

## Abstract

**Background & Aims:**

Bile acids (BAs) are major regulators of hepatic BA and lipid metabolism but their mechanisms of action in non-alcoholic fatty liver disease (NAFLD) are still poorly understood. Here we aimed to explore the molecular and biochemical mechanisms of ursodeoxycholic acid (UDCA) in modulating the cross-talk between liver and visceral white adipose tissue (vWAT) regarding BA and cholesterol metabolism and fatty acid/lipid partitioning in morbidly obese NAFLD patients.

**Methods:**

In this randomized controlled pharmacodynamic study, we analyzed serum, liver and vWAT samples from 40 well-matched morbidly obese patients receiving UDCA (20 mg/kg/day) or no treatment three weeks prior to bariatric surgery.

**Results:**

Short term UDCA administration stimulated BA synthesis by reducing circulating fibroblast growth factor 19 and farnesoid X receptor (FXR) activation, resulting in cholesterol 7α-hydroxylase induction mirrored by elevated C4 and 7α-hydroxycholesterol. Enhanced BA formation depleted hepatic and LDL-cholesterol with subsequent activation of the key enzyme of cholesterol synthesis 3-hydroxy-3-methylglutaryl-CoA reductase. Blunted FXR anti-lipogenic effects induced lipogenic stearoyl-CoA desaturase (SCD) in the liver, thereby increasing hepatic triglyceride content. In addition, induced SCD activity in vWAT shifted vWAT lipid metabolism towards generation of less toxic and more lipogenic monounsaturated fatty acids such as oleic acid.

**Conclusion:**

These data demonstrate that by exerting FXR-antagonistic effects, UDCA treatment in NAFLD patients strongly impacts on cholesterol and BA synthesis and induces neutral lipid accumulation in both liver and vWAT.

## Graphical abstract

**Figure:**
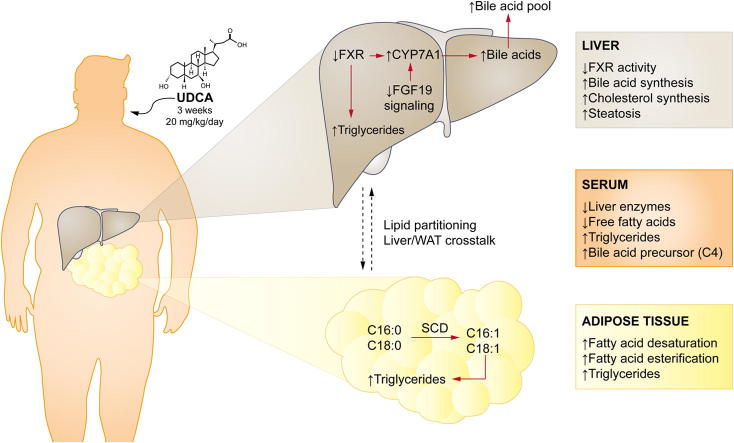

## Introduction

Obesity is a major risk factor for the development of type 2 diabetes, hypertension and dyslipidemia exerting adverse effects on the liver. The prevalence of non-alcoholic fatty liver disease (NAFLD), as hepatic manifestation and major complication of obesity and the metabolic syndrome, is dramatically rising and comprises a spectrum ranging from steatosis over non-alcoholic steatohepatitis (NASH) to advanced fibrosis/cirrhosis, ultimately leading to liver cancer. NAFLD is found in over two thirds of the obese population, regardless of diabetic status, and in more than 90% of morbidly obese individuals (>40 kg/m^2^ body mass index (BMI)); NASH is diagnosed in 19% and almost 50% of these individuals, respectively [1]. A key feature of NAFLD is the hepatic accumulation of triglycerides (TGs) and free cholesterol. Expansion of white adipose tissue (WAT) with increased lipolysis and flux of fatty acids (FAs) to the liver due to insulin resistance (IR) critically links WAT dysfunction to NAFLD/NASH development [2].

In addition to their detergent properties in lipid digestion, bile acids (BAs) serve as signaling molecules by activating dedicated receptors such as the nuclear farnesoid X receptor (FXR) in the liver and intestine, which impacts on BA and lipid metabolism [3], [4]. Upon FXR activation, BA homeostasis is maintained via a negative feedback loop decreasing expression of cholesterol 7α-hydroxylase (CYP7A1), the key enzyme in BA *de novo* synthesis, which mediates the conversion of cholesterol into BAs. Hepatic CYP7A1 is repressed by FXR-induced small heterodimer partner (SHP) and by fibroblast growth factor 19 (FGF19). In addition, FXR stimulation lowers triglyceridemia and hepatic TG deposition by reducing the expression of lipogenic genes and their regulators including sterol regulatory element-binding protein-1c (SREBP1c), fatty acid synthase (FASN) and stearoyl-CoA desaturase (SCD) [3]. Conversely, FXR deficiency in mice results in marked hypertriglyceridemia as a result of low apolipoprotein C-II and high apolipoprotein C-III, which reduces the interactions of chylomicrons and very low density lipoproteins (VLDL) with the lipoprotein lipase and their breakdown [5].

Ursodeoxycholic acid (UDCA) is currently used as ‘panacea’ for pharmacological treatment for a wide range of hepatobiliary disorders and has been shown to improve steatosis and inflammation in mice [6]. Taurine-conjugated UDCA reduced hepatic steatosis and enhanced insulin action in mouse liver, muscle and WAT [7] and enhanced hepatic and muscle insulin sensitivity in obese humans [8].

Clinical studies with UDCA in NAFLD have generated results raising questions about therapeutic mechanisms of BAs: While two randomized placebo-controlled trials did not show overall histological improvement including ballooning and inflammation [9], [10], a recent high-dose UDCA study attenuated hepatic IR [11]. Importantly, understanding the mechanism(s) of action of UDCA may be instrumental for the development of more effective BA-based therapies for NAFLD/NASH.

In the present study, we aimed to investigate potential effects and underlying mechanisms of short term UDCA exposure on the interplay between hepatic and visceral WAT (vWAT) metabolism by analyzing; (i) BA and cholesterol homeostasis; (ii) biliary transporter expression; and (iii) FA/lipid partitioning in morbidly obese patients with NAFLD/NASH. We herein uncover numerous mechanistically interrelated changes in serum parameters and mRNA expression patterns of genes involved in BA, cholesterol and lipid metabolism. Moreover, we provide a detailed lipidomic profile of liver and vWAT uncovering altered storing properties upon UDCA administration.

## Patients and methods

### Study population

Patients with morbid obesity (BMI >35 kg/m^2^) scheduled for laparoscopic Roux-en-Y gastric bypass surgery at Ersta Hospital, Stockholm, were asked to participate in a clinical pharmacodynamic study of the metabolic and molecular effects of UDCA. All candidates completed a detailed questionnaire about the patient’s history and life-style. A total of 40 well-matched patients were equally randomized by drawing lots to treatment with UDCA, 20 mg/kg/day, for three weeks (Ursofalk®, Dr. Falk, Freiburg, Germany; kind gift of MEDA, Stockholm, Sweden) or no medication (controls) before surgery. UDCA was administered open-label in two daily doses until the day before surgery, i.e. the first dose was given after drawing blood on day 1, the last dose in the evening before surgery on day 21. Liver, kidney, intestinal or metabolic diseases other than NAFLD/NASH (alanine aminotransferase (ALT)/aspartate aminotransferase (AST)/gamma-glutamyl transferase (γGT) <3 × ULN) were exclusion criteria, as well as the use of medications known to affect liver function and metabolism. Blood samples were taken in the fasting state at 8:00 AM, and tissue samples by ultrasound dissector during surgery. No day 21 serum samples were taken in the control group. Based on a widely accepted histological scoring system [12], the lesions of NAFLD were classified as fatty liver or steatohepatitis on liver biopsies after surgery by a board certified pathologist (C.L.).

All participants provided written informed consent. The study protocol (ClinicalTrials.gov NCT01548079) was approved the by Ethics Committee at Karolinska Institutet (Dnr 2008/2:3) and the Swedish Medical Products agency (EudraCT 2007-005531-28).

For more details, see Supplementary Materials and Methods.

## Results

### Patient characteristics

Out of 40 randomized patients, 19 finished per protocol in the UDCA and 18 in the control groups. Drop-outs were due to diarrhea in the UDCA, and pregnancy and minor intraoperative bleeding in the control groups. Gender, age, BMI, liver function tests and IR (estimated by HOMA-IR) did not differ between groups. Compliance to UDCA (>95% in each patient randomized to treatment) was confirmed by pill counts and UDCA measurements in serum. All participants had been instructed not to change their dietary habits, thus, BMI increased during the study period, both in UDCA (42.4 ± 5.1 kg/m^2^ to 43.2 ± 5.2 kg/m^2^, *p* <0.05) and control (40.6 ± 3.9 kg/m^2^ to 41.1 ± 3.7 kg/m^2^, *p* <0.05) groups. Interestingly, histological analysis revealed a higher steatosis grade (1.2 to 1.9, *p* <0.05) and thereby NAFLD activity score (NAS) (1.9 to 2.5, *p* <0.05) in the UDCA treated patients compared to untreated controls at the day of surgery. Baseline HOMA-IR classified 18/19 UDCA and 15/18 control patients as insulin resistant. Fasting glucose and HbA1c levels were normal in all patients.

### UDCA increased BA synthesis and cholesterol turnover

UDCA treatment resulted in reductions of serum AST, γGT, as well as free FA, total and LDL-cholesterol (LDL-C), whereas TGs increased (Table 1).

**Table 1:** **Serum parameters before and after UDCA treatment.**

LDL, low density lipoprotein; HDL, high density lipoprotein; n.s., not significant.

Upon UDCA, BAs increased 10-fold with UDCA enrichments in the range of recently reported peak concentrations in non-cholestatic subjects [13]. UDCA constituted 87.7 ± 3.7% of total BAs, in equal amounts unconjugated or glycine-conjugated (Supplementary Table 1). Of note, also the amounts of primary BAs chenodeoxycholic acid (CDCA) and cholic acid (CA) increased as their glycine-conjugates (Supplementary Table 1). Whereas CDCA elevations may have resulted from intestinal and/or hepatic epimerization of UDCA [14], the simultaneous increase of CA indicated enhanced *de novo* synthesis.

**Fig. 1:**
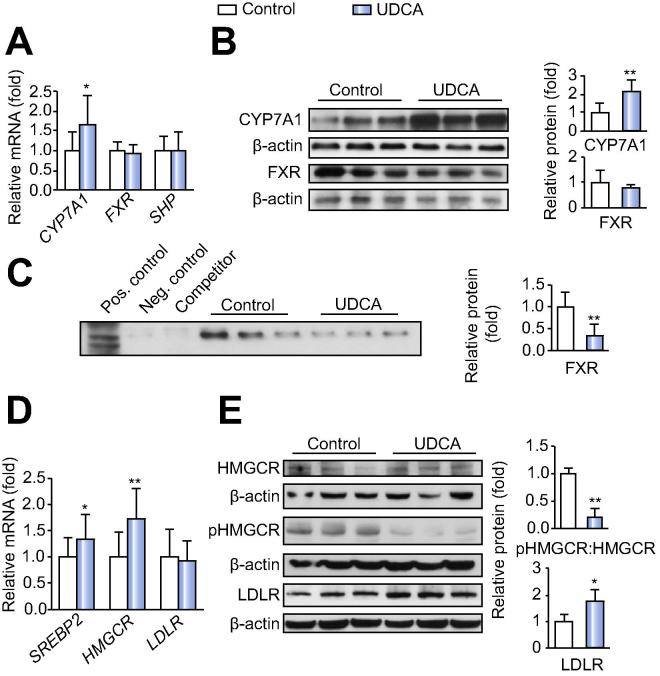
**UDCA alters key determinants of hepatic bile acid and cholesterol homeostasis.** (A) mRNA analysis of bile acid biosynthesis markers. Controls: n = 18; UDCA: n = 19. (B) Representative Western blots of CYP7A1, FXR and densitometry (all samples) of protein levels relative to β-actin. Controls: n = 7; UDCA: n = 6. (C) ABCD-assay indicating FXR activity. Controls: n = 7; UDCA: n = 6. (D) mRNA analysis of cholesterol biosynthesis markers. Controls: n = 18; UDCA: n = 19. (E) Representative Western blots of HMGCR, phosphorylation status of HMGCR (HMGCRp), LDLR and densitometry (all samples). Controls: n = 7; UDCA: n = 6. Mean values ± SD are expressed for all data. ^∗^*p* ⩽0.05, ^∗∗^*p* ⩽0.01 *vs.* control group.

We first focused on changes in BA metabolism. Serum BA precursors, 7α-hydroxy-cholesterol and 7α-hydroxy-4-cholesten-3-one (C4), were increased (Table 1) and mRNA and protein expression levels of CYP7A1 were higher in liver samples of UDCA treated patients compared to controls (Fig. 1A and B). Thus, BA biosynthesis was clearly stimulated upon UDCA. Notably, negative feedback regulation of BA homeostasis via hepatic FXR/SHP was not activated as reflected by unchanged SHP mRNA expression (Fig. 1A) and decreased formation of FXR/RXR DNA sequence complexes in the ABCD-assay (Fig. 1C). Rather, BA synthesis was enhanced via decreased circulating FGF19 (Table 1), the inhibitor of CYP7A1 [15]. Further nuclear receptors such as CAR and PXR remained unchanged on mRNA level upon UDCA (data not shown).

Enhanced BA synthesis should affect cholesterol turn-over. Indeed, we observed elevated hepatic mRNA levels of the transcriptional regulator *SREBP2* and its target, 3-hydroxy-3-methylglutaryl-CoA reductase (*HMGCR*), the rate-determining enzyme in cholesterol synthesis (Fig. 1D) [16]. This was further substantiated by decreased HMGCR-phosphorylation (Fig. 1E). UDCA treatment thus enhanced cholesterol synthesis as well. Nevertheless, steady hepatic cholesterol flux is suggested by unchanged mRNA expression of the ATP-binding cassette transporters ABCA1, ABCG1 mediating cholesterol efflux and the biliary cholesterol transporters ABCG5 and ABCG8 (data not shown). Notably, despite unchanged mRNA levels of the LDL-receptor (*LDLR*), increased LDLR protein expression (Fig. 1E) points towards increased cholesterol uptake from blood, which could explain the observed decreases in total and LDL-cholesterol upon UDCA treatment (Table 1).

Taken together, our results indicate that UDCA treatment induced BA synthesis fueled by hepatic cholesterol. The liver compensated cholesterol consumption via two pathways: i) the induction of *de novo* cholesterol synthesis via SREBP2/HMGCR; and ii) cholesterol uptake via LDLR.

### Highly enriched UDCA does not alter hepatobiliary transporter expression in NAFLD

Changes in BA flux through the liver may affect BA signaling and in turn, hepatic BA and lipid homeostasis. Therefore, we examined the effects of UDCA on hepatobiliary transporter expression. No differences between untreated or UDCA treated groups were observed in relation to RNA or protein expression of MRP2, MRP3, MDR3 and BSEP (Supplementary Fig. 1A–C). Notably, upregulation of MRP4 mRNA was not reflected by changes in protein expression (Supplementary Fig. 1A–C). Thus, UDCA did not affect hepatobiliary transporter expression including NTCP and OST-alpha/beta (data not shown) in morbidly obese patients, in contrast to non-obese gallstone patients [17].

### UDCA affects triglyceride and fatty acid partitioning in liver and visceral white adipose tissue

To examine the impact of UDCA on lipid and cholesterol partitioning along the liver-WAT axis, we performed tissue concentration measurements and lipid profiling.

**Fig. 2:**
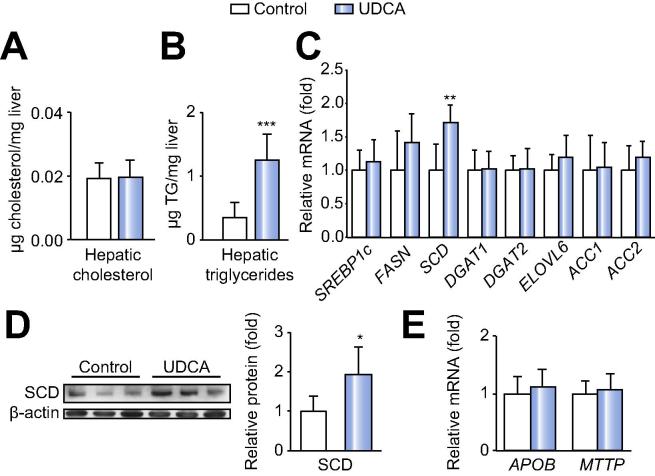
**UDCA increases hepatic triglyceride formation and modulates hepatic SCD expression.** (A) Hepatic cholesterol and (B) triglyceride concentrations. (C) Hepatic mRNA expression analysis of markers of lipid metabolism in morbidly obese NAFLD patients. Control: n = 18; UDCA: n = 19. (D) Representative Western blot and densitometry (all samples) of hepatic SCD relative to β-actin. Control: n = 7; UDCA: n = 6. (E) mRNA expression of APOB and MTTP in NAFLD patients. Control: n = 18; UDCA: n = 19. Mean values ± SD are expressed for all data. ^∗^*p* ⩽0.05, ^∗∗^*p* ⩽0.01 *vs.* control group.

Hepatic cholesterol content (Fig. 2A) did not differ in UDCA treated or untreated patients, despite changes in cholesterol synthesis and uptake proteins. However, we observed an increase in hepatic TG levels (Fig. 2B). FA profiling of the total liver FA pool revealed an overall accumulation of FA species such as myristic (MA, 14:0), palmitic (PA, 16:0), palmitoleic (16:1n7), stearic (SA, C18:0) and oleic acids (OA, 18:1n9), whereas free FA species were unaltered upon UDCA treatment (Table 2). We thus determined the expression of lipid metabolism regulatory genes. SCD, the enzyme catalyzing the formation of monounsaturated FAs such as OA [18], was induced on mRNA and protein levels upon UDCA, whereas expression of other lipogenic genes such as SREBP1c, FASN and ACC1/2 remained unaltered (Fig. 2C and D). Moreover, the microsomal TG transfer protein (MTTP) and apolipoprotein B (ApoB), which are involved in VLDL export, did not differ between the groups (Fig. 2E). Thus, UDCA likely stimulated hepatic FA deposition as TG via hepatic SCD upregulation due to the absence of FXR-mediated anti-lipogenic effects.

**Table 2:** **Changes of fatty acid species in liver and vWAT upon UDCA.**

Total fatty acid (TFA) and free fatty acid (FFA) species are relative to internal standards. n.s., not significant.

**Fig. 3:**
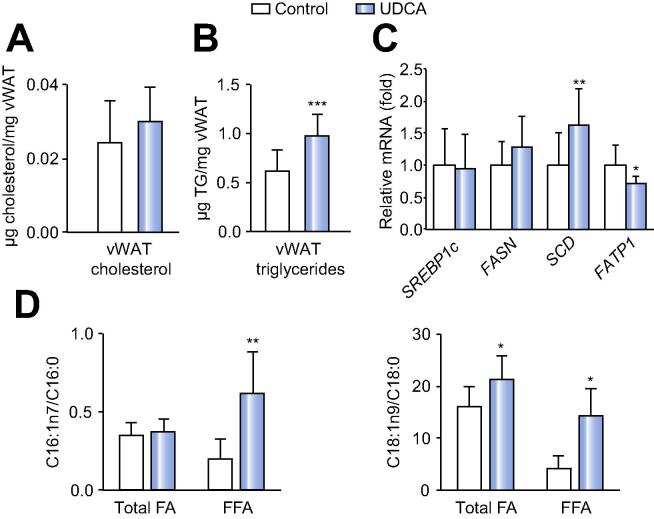
**UDCA induces triglyceride formation and lipogenic gene expression in visceral white adipose tissue (vWAT).** (A) Measurement of cholesterol and (B) triglyceride content in vWAT. (C) Relative mRNA expression of markers of *de novo* lipogenesis and fatty acid (FA) transport in vWAT. (D) SCD activity calculated according to C16:1/C16:0 and C18:1/C18:0 concentrations in total and free FA obtained from lipid profiling. Control: n = 16; UDCA: n = 14. Mean values ± SD are expressed for all data. ^∗^*p* ⩽0.05, ^∗∗^*p* ⩽0.01, ^∗∗∗^*p* ⩽0.001 *vs.* control group.

In vWAT of UDCA treated patients an increased TG load, again without changes in cholesterol levels, was found (Fig. 3A and B). Notably, UDCA treatment resulted in enrichment of OA in the total FA fraction (Table 2), in line with the upregulation of *SCD* mRNA (Fig. 3C). As observed in liver, expression of other lipogenic genes such as FASN and SREBP1c did not differ between the groups in vWAT (Fig. 3C).

Conversely, analysis of free FAs in vWAT revealed decreased levels of free OA, together with MA, PA and SA upon UDCA treatment (Table 2). Consistently, lower free FA concentrations were also detected in the serum (Table 1). Additionally, FA transport protein (*FATP1*) was reduced on mRNA level (Fig. 3C), which in part could be attributed to restricted plasma free FA availability (Table 1). The small amounts of protein in vWAT, limited our expression analysis to mRNA levels. However, as a measure of SCD activity, the ratio of unsaturated and saturated C16 and C18 was calculated from total and free FAs. Except for C16 total FA-ratio, desaturation processes and hence SCD activity were induced upon UDCA treatment (Fig. 3D).

Since OA is the preferential substrate for TG storage and prevents lipotoxicity [19], our data suggests a shift in lipid metabolism towards FA incorporation into visceral lipid droplets, which was not related to changes in anti-lipolytic FGF21 serum levels (Table 1). Unfortunately we could not address potential UDCA effects on vWAT lipolysis due to virtually absent vWAT enzymatic activity (data not shown).

## Discussion

In this pharmacodynamic study we analyzed liver tissue and vWAT obtained during bariatric surgery from short term UDCA treated and untreated morbidly obese NAFLD patients leading to decreased FXR activity, stimulated cholesterol and BA synthesis, hepatic lipid accumulation and altered lipid conversion in vWAT. Here, we provide a comprehensive evaluation of mechanistically interrelated metabolic alterations associated with UDCA treatment, which may be most relevant for the development of more effective BA mimetics for the treatment of NAFLD.

Stimulated BA synthesis during UDCA treatment, first described by Einarsson *et al.* [20], was later not consistently confirmed [21]. Furthermore, previous studies showed a considerable increase in cholesterol and BA synthesis in obesity [22]. We found that highly enriched UDCA enhances *de novo* BA and cholesterol synthesis via induction of several BA and cholesterol synthetic markers and enzymes such as C4, 7α-hydroxycholesterol and CYP7A1 (for BAs) and hepatic SREBP2 and HMGCR (for cholesterol) in morbid obesity. Since enhanced cholesterol synthesis in turn fueled stimulated BA formation, no net effect on hepatic cholesterol levels was observed. BAs suppress CYP7A1 via hepatic FXR/SHP and intestinal FXR/FGF19 signaling pathways [23]. However, UDCA, which constituted almost 90% of BAs in our patients, has only low affinity for and hence no agonistic activity on FXR [24], [25], [26], [27], [28]. Indeed, ABCD-assays suggest a decreased interaction of the FXR/RXR heterodimer with its DNA binding sequence and hence reduced FXR activation upon UDCA. Notably, this mechanism distinguishes UDCA from highly potent FXR agonist obeticholic acid recently proven beneficial in NASH [29], [30]. Moreover, our data suggest FXR-mediated reduction of intestinal FGF19 synthesis and secretion indicated by decreased FGF19 serum concentrations, which are inverse to hepatic CYP7A1 expression levels. This further supports the concept that UDCA abolishes endogenous FXR effects to an extent even reaching FXR-antagonistic properties, reflected by increased mRNA and protein expression of CYP7A1; by increased serum levels of BA precursors 7α-hydroxycholesterol and C4, and primary BAs; as well as by lowered serum FGF19 concentrations, decreased FXR/RXR DNA binding activities and blunted FXR mediated anti-lipogenic actions. While such effects may be beneficial for cholesterol catabolism, FXR-antagonistic properties could also explain UDCA’s limited clinical efficiency [9], [10], [11] in comparison to FXR agonist obeticholic acid, which is associated with increased insulin sensitivity upon 6 weeks treatment in diabetic NAFLD patients and improved liver histology but not IR upon 72 weeks of administration in NASH patients [29], [30]. Interestingly, UDCA, circulating in the enterohepatic system, had no impact on hepatobiliary transporter expression in morbidly obese patients, in contrast to a previous study in non-obese gallstone patients [17], which might be related to approximately tenfold higher total BA concentrations in the current study cohort.

Recently, upregulation of SREBP2 and HMGCR resulting in increased serum LDL-C, and accumulating hepatic free cholesterol and LDL-C was related to the pathogenesis and progression of human NAFLD [31]. Despite enhanced cholesterol *de novo* synthesis, our UDCA treated cohort featured decreased serum total- and LDL-C concentrations. Thus, to meet the demands of induced BA generation due to blunted FXR activation, cholesterol was also mobilized from the periphery via LDLR since cholesterol catabolism was compensated only in part by *de novo* synthesis.

Induced de-esterification of cholesterol esters via neutral cholesterol ester hydrolase (nCEH) has been linked to human NAFLD [31]. However, our data suggest that short term UDCA treatment does not affect hepatic free cholesterol and cholesterol ester homeostasis, since expression of both acetyl-CoA acetyltransferase 2 and nCEH, the enzymes regulating cholesterol esterification and de-esterification [32], was similar in both groups (data not shown). Based on these results, we conclude that *de novo* synthesized cholesterol is directed towards BA synthesis rather than cholesterol storage, consistent with unchanged hepatic cholesterol levels.

Since WAT and liver lipid metabolism are tightly linked, disturbed energy homeostasis in vWAT (e.g. increased FA flux to the liver as a result of IR) may exert deleterious effects on hepatic FA turn-over [33]. Here we show that short term UDCA administration, by replacing hydrophobic FXR-agonistic BAs and thereby reducing FXR anti-lipogenic effects, impacts on lipid conversion processes via elevated hepatic SCD expression. Reduction of serum free FAs and induction of serum TGs resemble lipid conversion events in vWAT and might be a consequence of altered FA conversion processes via upregulated SCD expression in both liver and vWAT. Despite decreased FXR signaling, direct UDCA effects on SREBP-1c, a known FXR target regulating FASN [3], are missing. Unchanged SREBP-1c expression might be explained by the gene’s high sensitivity to insulin [34], which did not change within 21 days of UDCA treatment (data not shown). Thus, we hypothesize that absent SREBP-1c repression via FXR might represent one of the reasons for UDCA mediated induction of hepatic lipid accumulation. Moreover, blunted FXR-mediated anti-lipogenic effects are indicated by elevated histology-proven steatosis scores and increased hepatic TGs along with induced FA species in the total FA pool upon UDCA. In contrast to other studies showing unaltered NAS, in which obese patients were encouraged to lose weight [9], [11], our participants were instructed not to change their dietary habits – possibly further promoting the adverse liver histology outcome. Besides these potentially harmful properties, these changes could also represent a hepatic mechanism counteracting injury from exogenous lipid overflow, known to mediate liver injury and apoptosis. Notably, conditions when SCD activity was induced and monounsaturated FAs and TGs constituted the major lipid species, were shown to attenuate lipotoxicity [19].

Lipid profiling of vWAT indicates that a combination of increased OA (18:1n9) in the total FA pool and a corresponding reduction of several lipid species in the free FA pool may contribute to a potentially beneficial shift towards improved lipid storage and reduced lipotoxicity upon UDCA. Increased expression of SCD appears to be the mechanism behind elevated concentrations of monounsaturated OA (18:1n9) in the total FA pool. OA (18:1n9) accumulation along with the observation that, except SCD, other lipogenic genes did not differ between the groups, suggests an increase in FA synthesis, which was not detected via mRNA analysis but by the accumulation of final products. The conversion of PA (16:0) and SA (18:0) deriving from the free FA pool and representing preferential substrates for SCD [35], could explain both their reduction in the free FA pool and the elevation of OA (18:1n9) loads in the total FA pool. Due to TG accumulation in vWAT, we hypothesize that expanded OA (18:1n9) levels derived from the free FA pool might be preferentially stored as TGs in lipid droplets in vWAT and thereby prevent lipotoxic effects of other potentially harmful lipid species.

Elevated SCD activity in vWAT could be explained by surplus of substrate for lipogenesis from hepatic lipid spill-over into the circulation. However, the exact mechanism behind SCD activation in vWAT upon short term UDCA treatment awaits further experimental elucidation. Apart from increased OA (18:1n9), we identified a decrease of vaccenic acid (VA, 18:1n11) in the total FA pool, which is mainly provided by ruminant meat and dairy products [36] and might be caused by less efficient micelle formation and dietary lipid absorption in the UDCA treated cohort [37].

Besides its function as a FA transport protein, FATP1 possesses acyl-CoA synthetase activity, generating substrates for β-oxidation [38]. Therefore, the reduction of FATP1 mRNA levels in vWAT suggests blunted visceral long chain free FA uptake and decreased very long chain FA activation. However, genes linked to β-oxidation such as CPT1α and AOX, did not differ between groups in liver or vWAT (data not shown). Of note, loss of FATP1 function in mice redistributes lipids from WAT and muscle towards the liver leading to hepatic TG accumulation [39]. This might reflect an additional mechanism inducing hepatic TG accumulation upon UDCA. Induction of alternative FA transport proteins in vWAT such as CD36 and FATP4, potentially restoring FA transport homeostasis, was not observed (data not shown).

The limitations of our study are the lack of placebo, of biopsies before UDCA treatment and of feces sampling for BA measurements. Due to restricted biopsy material, distinct investigations are based on mRNA analysis; however key findings are validated on protein level and are also supported by different biochemical methods.

**Fig. 4:**
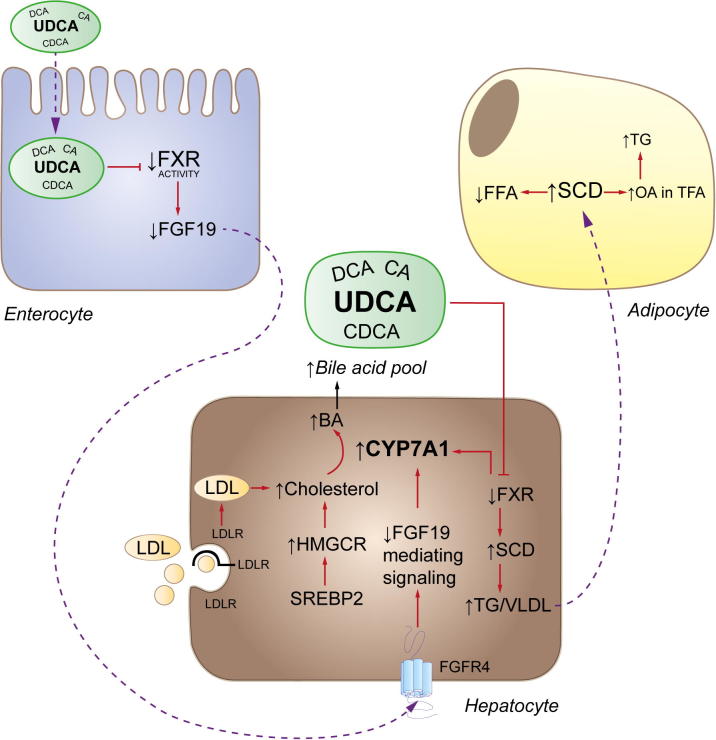
**Overview of UDCA mediated effects in morbid obesity.** UDCA decreases intestinal FXR activation. Reduced circulating FGF19 levels induce CYP7A1 activity and BA formation. Compensation of stimulated cholesterol conversion into BAs is regulated via LDL-C import and cholesterol *de novo* biosynthesis. Elevated UDCA concentrations repress hepatic FXR activity, thereby decreasing FXR mediated anti-lipogenic effects resulting in induced SCD expression and TG accumulation. Hepatic TG-overload is secreted into the circulation and stored in vWAT. Delivered free FAs (FFA) are converted into OA in the total fatty acid (TFA) pool and TG formation occurs due to elevated SCD activity in vWAT. (This figure appears in colour on the web.)

In summary, this unique pharmacodynamic study in morbid obesity sheds new light on the therapeutic mechanisms and potential limitations of UDCA in liver and vWAT and provides novel mechanistic insights in the regulation of metabolic pathways in a population of uncomplicated NAFLD outlined in Fig. 4. Short term UDCA treatment, by blunting FXR activity, increases BA generation, thereby increasingly utilizing hepatic cholesterol and subsequently inducing cholesterol *de novo* biosynthesis and LDLR expression. Deficient FXR activation also increases SCD mediated hepatic TG formation. Understanding SCD activation and the cytoprotective mechanism behind UDCA action, counteracting accumulation of lipotoxic FA species in vWAT, may facilitate the development of novel BA-based therapeutic strategies and/or expanding the use of UDCA.

## Financial support

Research was funded by the 10.13039/501100002428Austrian Science Fund (F3008, F3517 and the DK-MCD project W1226) and the European Community’s Seventh Framework Program (FP7/2007-2013) under grant agreement HEALTH-F2-2009-241762 for the project FLIP (to MT), grants from the 10.13039/501100004359Swedish Research Council (K2005-72X-04793-30A) and the Swedish Medical Association (to HUM), grants by the 10.13039/100007436Erling-Persson Family Foundation (to AT) and grants through the regional agreement on medical training and clinical research (ALF) between 10.13039/501100004348Stockholm County Council and 10.13039/501100004047Karolinska Institutet (to AT, HUM).

## Conflict of interest

The authors who have taken part in this study have declared a relationship with the manufacturers of the drugs involved.

## Authors’ contributions

The corresponding authors developed the study concept and design, critically reviewed the data and decided the content of the manuscript. All other authors acquired and/or analyzed data, performed statistical analysis and/or provided technical or material support and/or wrote and/or critically reviewed the manuscript. All authors finally decided the content of the manuscript.

## Supplementary data

Supplementary data

Supplementary data CONSORT 2010 Checklist.

Supplementary data CONSORT 2010 Flow Diagram.
