# Supplementary material for: Ursodeoxycholic acid exerts farnesoid X receptor-antagonistic effects on bile acid and lipid metabolism in morbid obesity
Source: J Hepatol. 2015 Jun;62(6):1398–404. doi: 10.1016/j.jhep.2014.12.034 (PMC4451470; doi:10.1016/j.jhep.2014.12.034)
Supplement: Supplementary data [file mmc1.pdf]

Supplementary data to:

Ursodeoxycholic acid exerts farnesoid X receptor-antagonistic effects on bile acid and lipid metabolism in morbid obesity

Michaela Mueller, Anders Thorell, Thierry Claudel, Pooja Jha, Harald Koefeler, Carolin Lackner, Bastian Hoesel, Guenter Fauler, Tatjana Stojakovic, Curt Einarsson, Hanns-Ulrich Marschall[#], Michael Trauner[#]

[#]Shared senior authorship and correspondence

Table of Contents

Supplementary Materials and Methods 2
- Serum clinical chemistry, C4, and FGF19/21 2
- Serum bile acids 2
- Serum sterols 2
- Messenger RNA expression analysis 3
- Protein extraction and western blot analysis 3
- Avidin biotin complex DNA-assay 3
- Tissue triglyceride and cholesterol determination 4
- Adipose tissue and liver lipid profiling 4
  - *GC-NICI/MS of free fatty acids* 4
  - *GC-EI/MS of total fatty acids (free and esterified)* 5
  - *GCMS of free and total fatty acids* 5
- Statistical analysis 6

Supplementary Table 7
Supplementary Figure 8
References 9

Supplementary Materials and Methods

Serum clinical chemistry, C4, and FGF19/21

Cholesterol fractions (total, HDL and LDL), TGs, as well as liver and kidney function tests were analyzed using standard clinical biochemical procedures. Serum 7α-hydroxy-4-cholesten-3-one (C4) was measured by HPLC in comparison to the internal standard 7β-hydroxy-4-cholesten-3-one (Steraloids, RI, USA) [1], Serum FGF19 and FGF21 were measured by ELISA according to the manufacturer's handbook (R&D Systems, Oxon, UK). All samples were taken in the fasting state at 8:00 AM.

Serum bile acids

Serum bile acids were analyzed by ultra-performance liquid chromatography-tandem mass spectrometry (UPLC-MS/MS) after adding D_4-isotope-labeled internal standards according to recently described conditions [2]. Stable isotope-labeled reference compounds (D_4-CA, D_4-UDCA, D_4-LCA, D_4- GCA, D_4-GUDCA, D_4-GLCA) were from Qmx laboratories (Essex, UK), unlabeled unconjugated (LCA, DCA, CDCA, HDCA, UDCA, CA, HCA) and glycine- or taurine-conjugated reference bile acids (GLCA, GDCA, GCDCA, GCA, GUDCA, TLCA, TDCA, TCDCA, TCA, TUDCA) were from Sigma-Aldrich (St Louis, MO, USA) and Steraloids (RI, USA).

Serum sterols

Serum 7-hydroxysterols were analyzed by gas-chromatography-mass spectrometry (GCMS) as previously described [3].

Messenger RNA expression analysis

Total RNA isolation, complementary DNA synthesis, quantitative real-time reverse-transcription polymerase chain reaction (qRT-PCR) and messenger RNA (mRNA) expression analysis from liver and vWAT were performed as previously described [4]. mRNA levels were normalized to the housekeeping genes 36b4 or 18S, which did not vary between the groups. Oligonucleotide sequences are available upon request.

Protein extraction and western blot analysis

Analysis of hepatobiliary transport proteins and cytosolic and nuclear fractions was performed via Western blotting as previously described [5, 6]. Blots were probed with antibodies specific to the protein of interest as primary antibodies. The secondary antibodies were peroxidase-conjugated. Commercial kits were used for the detection of the proteins (Pierce ECL Plus Western Blotting Substrate, Thermo Scientific, USA). For quantification of protein expression, blots were scanned and analysed using ImageJ http.//imagej.nih.gov/ij/index.html) Protein signals were normalized to β-actin.

Avidin biotin complex DNA-assay

The avidin, biotin, complex DNA (ABCD)-Assay was used to immobilize the FXR/RXR heterodimer/DNA-complex via binding of the biotinylated FXR/RXR target DNA sequence to streptavidin coated beads. Detailed assay information is available in Baumann S. et al [7]. Sequences for analysis are available upon request.

Tissue triglyceride and cholesterol determination

Frozen tissue samples were extracted according to Folch [8]. For lipid and cholesterol extraction 60-80 mg frozen tissue were homogenized in methanol and extracted with choloroform and glacial acetic acid (chloroform:methanol:glacial acetic acid, 66:33:1, v/v/v). Water was added for phase separation. Dried lipids were dissolved under sonication in 0.1% Triton-X 100. TG and cholesterol concentrations were determined using commercial kits (Diagnostic Systems International, Holzheim, Germany for triglyceride measurement and Abcam, Cambridge, United Kingdom for cholesterol measurement).

Adipose tissue and liver lipid profiling

GC-NICI/MS of free fatty acids

Gas chromatography-negative ion chemical ionisation mass spectrometry (GC-NICI/MS) was used for profiling of esterified and non-esterified fatty acids (total FAs) About 50 mg of liver or vWAT were homogenized in 2.5 ml of methyl-tert-butylether (MTBE) and 0.7 ml of methanol. Each sample was spiked immediately with 4.5 nmol of FA 15:0 and 1.8 µmol FA 17:0 as internal standards. Then, lipids were extracted according to Matyash et al. [9]. Lipid extracts were dried and dissolved in 50 µl of a pentafluorobenzyl bromide solution (3.4 % in acetonitrile) and 10 µl of N, N-diisopropyl ethanolamine. After 10 min of incubation at room temperature samples were evaporated under a stream of nitrogen and suspended in 50 µl of hexane.

A Trace-DSQ GC-MS (Thermo Scientific, Austin, TX) equipped with a TR-FAME 30m column was used in splitless mode with 1 ml/min helium as carrier gas and 300°C injector temperature. The initial oven temperature of 150°C was held for 1min and then increased to 200°C at a rate of 25°C/min followed by 325°C at a rate of

12.5°C/min and held for 2min. The mass spectrometer was run in negative ion chemical ionization (NICI) mode. Fatty acids were detected in full scan as carboxylates after loss of the pentafluoro benzyl moiety. Methane was used as CI gas. Source temperature was set to 250°C and the transfer line temperature to 330°C. Quantitation of fatty acids was performed by correlating integrated areas of fatty acids versus the integrated area of FA 15:0.

GC-EI/MS of total fatty acids (free and esterified)

Dried lipid extracts from above were suspended in 1 ml methanol NaOH. After 10 min incubation at 80°C, samples were cooled for 5 min on ice. Then, 1 ml BF3 was added and samples were incubated for 10 min at 80°C. Fatty acid methyl esters were extracted with 1 ml saturated NaCl and 2 ml hexane. The hexane phase was dried and methyl esters dissolved in 1.5 ml hexane. A Trace-DSQ GC-MS equipped with a TR-FAME 30m column was used for analysis. Helium was used as carrier gas at a flow of 1.3 ml/min, in split mode, at 250°C injector temperature. The initial oven temperature of 150°C was held for 0.5 min and then the temperature was increased to 180°C at a rate of 10°C/min. This was followed by a further increase to 190°C at a rate of 0.5°C/min and then increased to 250°C at a rate of 40°C/min and kept for 3 min. The mass spectrometer was run in electron impact mode and fatty acids were detected in full scan of m/z 80 - 400. Source temperature was set to 250°C and the transfer line temperature to 200°C. Peak areas for FAs were calculated by XcaliburQuanBrowser and related to FA 17:0 internal standard peak areas.

GCMS of free and total fatty acids

Gas chromatography-mass spectrometry (GCMS) was used for profiling the free FA pool and the total FA pool (including esterified and free FAs) as described .

Statistical analysis

Data are expressed as mean values ± standard deviation (SD). Differences were calculated with Mann-Whitney-U-Test analyzing unequally distributed parameters using the SigmaStat® statistic program (Jandel Scientific, San Rafael, CA. USA). A p value of < 0.05 was considered significant.

Supplementary Table

Table S1. Serum Bile Acid Before and After UDCA Treatment

BA [µM]	UDCA Day 1	UDCA Day 21	P-Value
UDCA	0.13 ± 0.11	5.79 ± 3.73	p<0.001
CA	0.13 ± 0.18	0.14 ± 0.14	n.s.
DCA	0.43 ± 0.31	0.31 ± 0.17	n.s.
CDCA	0.23 ± 0.23	0.35 ± 0.17	p<0.01
LCA	0.03 ± 0.01	0.03 ± 0.03	n.s.
T-UDCA	0.02 ± 0.02	0.21 ± 0.20	p<0.001
T-CA	0.04 ± 0.03	0.06 ± 0.05	n.s.
T-DCA	0.06 ± 0.07	0.06 ± 0.06	n.s.
T-CDCA	0.08 ± 0.08	0.10 ± 0.09	n.s.
T-LCA	0.00 ± 0.00	0.00 ± 0.00	n.s.
G-UDCA	0.12 ± 0.19	8.79 ± 7.66	p<0.001
G-CA	0.18 ± 0.18	0.47 ± 0.56	p<0.01
G-DCA	0.17 ± 0.2	0.29 ± 0.36	n.s.
G-CDCA	0.48 ± 0.68	1.20 ± 1.41	p<0.01
G-LCA	0.03 ± 0.01	0.05 ± 0.05	n.s.
TOTAL	1.89 ± 0.85	19.5 ± 12.2	p<0.001

UDCA, ursodeoxycholic acid; CA, cholic acid; DCA, deoxycholic acid; CDCA chenodeoxycholic acid; LCA, lithocholic acid; T, tauro-; G, glyco-; n.s., not significant.

Supplementary Figure

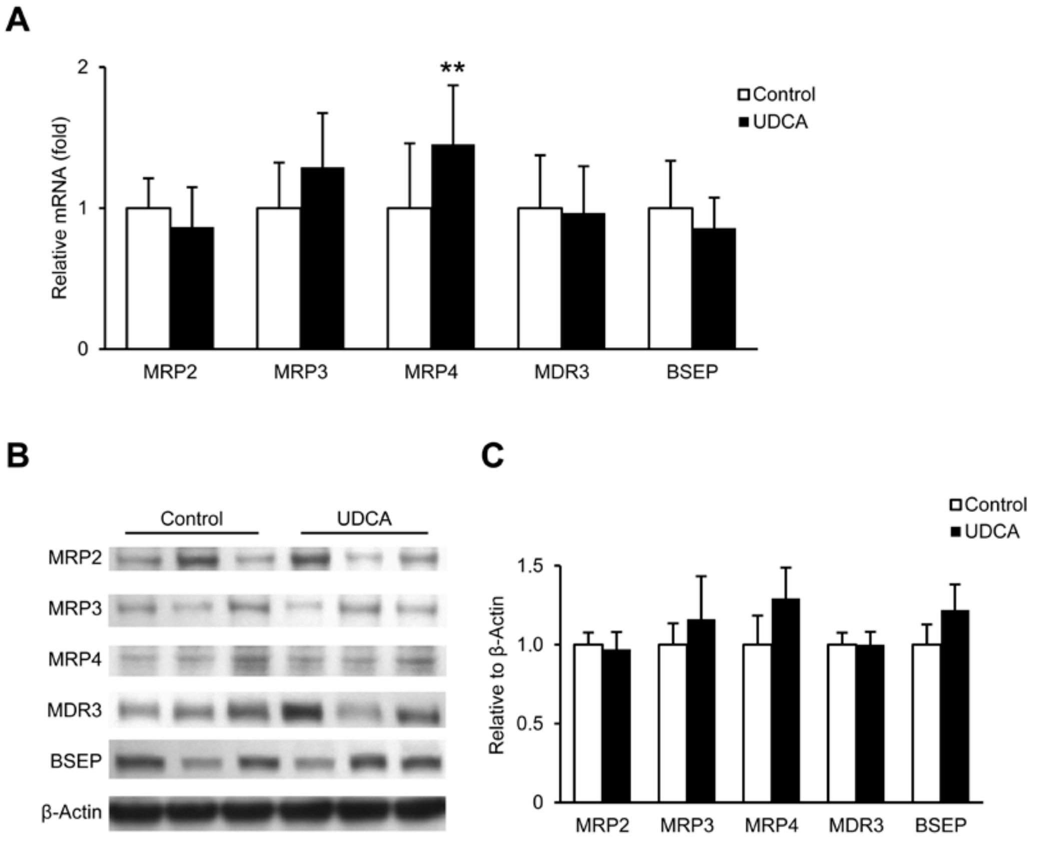

Fig. S1. Hepatobiliary ABC transporter expression remains unchanged upon highly enriched UDCA treatment. (A) Gene expression analysis of key hepatobiliary ABC transporters in morbidly obese NAFLD patients via qRT-PCR. Controls: n=18; UDCA: n=19. (B) Immunoblotting of hepatobiliary transporters of steatotic livers. Controls: n=7; UDCA: n=6. (C) Densitometry of protein levels (all samples). Control: n=7; UDCA: n=6. Mean values ± SD are expressed for all data. *p<0.05 versus control group.

References

[1] Galman C, Arvidsson I, Angelin B, Rudling M. Monitoring hepatic cholesterol 7alpha-hydroxylase activity by assay of the stable bile acid intermediate 7alpha-hydroxy-4-cholesten-3-one in peripheral blood. J Lipid Res 2003;44:859-866.

[2] Sayin SI, Wahlstrom A, Felin J, Jantti S, Marschall HU, Bamberg K, et al. Gut microbiota regulates bile acid metabolism by reducing the levels of tauro-beta-muricholic acid, a naturally occurring FXR antagonist. Cell Metab 2013;17:225-235.

[3] Stojakovic T, Putz-Bankuti C, Fauler G, Scharnagl H, Wagner M, Stadlbauer V, et al. Atorvastatin in patients with primary biliary cirrhosis and incomplete biochemical response to ursodeoxycholic acid. Hepatology 2007;46:776-784.

[4] Wagner M, Fickert P, Zollner G, Fuchsbichler A, Silbert D, Tsybrovskyy O, et al. Role of farnesoid X receptor in determining hepatic ABC transporter expression and liver injury in bile duct-ligated mice. Gastroenterology 2003;125:825-838.

[5] Marschall HU, Wagner M, Zollner G, Fickert P, Diczfalusy U, Gumhold J, et al. Complementary stimulation of hepatobiliary transport and detoxification systems by rifampicin and ursodeoxycholic acid in humans. Gastroenterology 2005;129:476-485.

[6] Fuchs CD, Claudel T, Kumari P, Haemmerle G, Pollheimer MJ, Stojakovic T, et al. Absence of adipose triglyceride lipase protects from hepatic endoplasmic reticulum stress in mice. Hepatology 2012;56:270-280.

[7] Baumann S, Dostert A, Novac N, Bauer A, Schmid W, Fas SC, et al. Glucocorticoids inhibit activation-induced cell death (AICD) via direct DNA-dependent repression of the CD95 ligand gene by a glucocorticoid receptor dimer. Blood 2005;106:617-625.

[8] Folch J, Lees M, Sloane Stanley GH. A simple method for the isolation and purification of total lipides from animal tissues. J Biol Chem 1957;226:497-509.

[9] Matyash V, Liebisch G, Kurzchalia TV, Shevchenko A, Schwudke D. Lipid extraction by methyl-tert-butyl ether for high-throughput lipidomics. J Lipid Res 2008;49:1137-1146.
