# Supplementary figures and images for: Ursodeoxycholic acid exerts farnesoid X receptor-antagonistic effects on bile acid and lipid metabolism in morbid obesity
Source: J Hepatol. 2015 Jun;62(6):1398–404. doi: 10.1016/j.jhep.2014.12.034 (PMC4451470; doi:10.1016/j.jhep.2014.12.034)

## CONSORT 2010 Flow Diagram

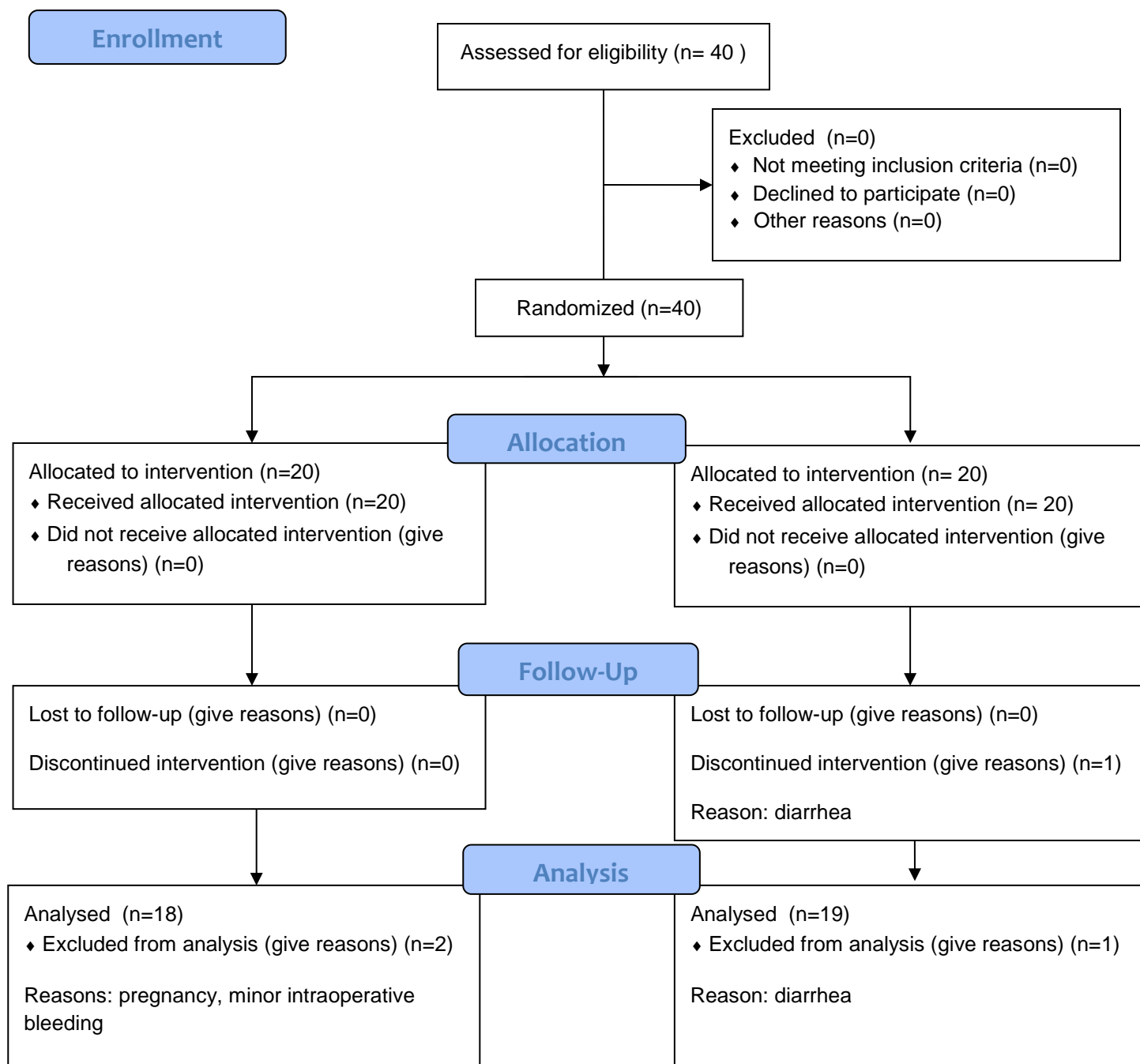

Supplement: Supplementary data — CONSORT 2010 Flow Diagram. [file mmc3.pdf]
